# Supplementary material for: miRNA-Mediated Epigenetic Regulation of Treatment Response in RA Patients—A Systematic Review
Source: Int J Mol Sci. 2022 Oct 27;23(21):12989. doi: 10.3390/ijms232112989 (PMC9657910; doi:10.3390/ijms232112989)
Supplement: Supplementary file 1 [file ijms-23-12989-s001.zip › ijms-1940483-supplementary.pdf]

Supplementary Materials:

Table S1. Bibliographic search

Pubmed	("arthritis, rheumatoid"[MeSH Terms] OR ("Arthritis"[All Fields] AND "Rheumatoid"[All Fields]) OR "rheumatoid arthritis"[All Fields] OR ("Rheumatoid"[All Fields] AND "Arthritis"[All Fields]) OR "rheumatoid arthritis"[Title/Abstract]) AND ("microrna s"[All Fields] OR "micrornas"[MeSH Terms] OR "micrornas"[All Fields] OR "microrna"[All Fields] OR ("micrornas"[MeSH Terms] OR "micrornas"[All Fields] OR "miRNA"[All Fields] OR "mirnas"[All Fields] OR "mirna s"[All Fields]) OR ("micrornas"[MeSH Terms] OR "micrornas"[All Fields] OR ("Micro"[All Fields] AND "RNA"[All Fields]) OR "micro rna"[All Fields]) OR ("micrornas"[MeSH Terms] OR "micrornas"[All Fields] OR ("RNA"[All Fields] AND "Micro"[All Fields]) OR "rna micro"[All Fields]) OR ("micrornas"[MeSH Terms] OR "micrornas"[All Fields] OR "miRNA"[All Fields] OR "mirnas"[All Fields] OR "mirna s"[All Fields]) OR ("micrornas"[MeSH Terms] OR "micrornas"[All Fields] OR ("Primary"[All Fields] AND "microrna"[All Fields]) OR "primary microrna"[All Fields]) OR ("micrornas"[MeSH Terms] OR "micrornas"[All Fields] OR ("microrna"[All Fields] AND "Primary"[All Fields]) OR "microrna primary"[All Fields]) OR ("micrornas"[MeSH Terms] OR "micrornas"[All Fields] OR ("Primary"[All Fields] AND "miRNA"[All Fields]) OR "primary mirna"[All Fields]) OR ("micrornas"[MeSH Terms] OR "micrornas"[All Fields] OR ("miRNA"[All Fields] AND "Primary"[All Fields]) OR "mirna primary"[All Fields]) OR ("micrornas"[MeSH Terms] OR "micrornas"[All Fields] OR ("pri"[All Fields] AND "miRNA"[All Fields]) OR "pri-miRNA"[All Fields]) OR ("micrornas"[MeSH Terms] OR "micrornas"[All Fields] OR ("pri"[All Fields] AND "miRNA"[All Fields]) OR "pri-miRNA"[All Fields]) OR ("micrornas"[MeSH Terms] OR "micrornas"[All Fields] OR ("RNA"[All Fields] AND "Small"[All Fields] AND "Temporal"[All Fields])) OR ("micrornas"[MeSH Terms] OR "micrornas"[All Fields] OR ("Temporal"[All Fields] AND "RNA"[All Fields] AND "Small"[All Fields])) OR ("micrornas"[MeSH Terms] OR "micrornas"[All Fields] OR "stRNA"[All Fields] OR "strnas"[All Fields]) OR ("micrornas"[MeSH Terms] OR "micrornas"[All Fields] OR ("Small"[All Fields] AND "Temporal"[All Fields] AND "RNA"[All Fields]) OR "small temporal rna"[All Fields]) OR ("micrornas"[MeSH Terms] OR "micrornas"[All Fields] OR ("pre"[All Fields] AND "miRNA"[All Fields]) OR "pre-miRNA"[All Fields]) OR ("micrornas"[MeSH Terms] OR "micrornas"[All Fields] OR ("pre"[All Fields] AND "miRNA"[All Fields]) OR "pre-miRNA"[All Fields]) OR "pre-miRNA"[Title/Abstract] OR "pre-miRNA"[Title/Abstract] OR

	"small temporal rna"[Title/Abstract] OR "stRNA"[Title/Abstract] OR (("Temporal"[All Fields] OR "temporally"[All Fields] OR "temporals"[All Fields]) AND "rna small"[Title/Abstract]) OR (("Temporal"[All Fields] OR "temporally"[All Fields] OR "temporals"[All Fields]) AND "rna small"[Title/Abstract]) OR (("RNA"[MeSH Terms] OR "RNA"[All Fields]) AND "small temporal"[Title/Abstract]) OR "pri-miRNA"[Title/Abstract] OR "pri-miRNA"[Title/Abstract] OR "mirna primary"[Title/Abstract] OR "primary mirna"[Title/Abstract] OR "miRNA"[Title/Abstract] OR "rna micro"[Title/Abstract] OR "micro rna"[Title/Abstract]) AND ("genetic variation"[MeSH Terms] OR ("Genetic"[All Fields] AND "Variation"[All Fields]) OR "genetic variation"[All Fields] OR ("Genetic"[All Fields] AND "Variations"[All Fields]) OR "genetic variations"[All Fields] OR "genetic variations"[Title/Abstract] OR ("genetic variation"[MeSH Terms] OR ("Genetic"[All Fields] AND "Variation"[All Fields]) OR "genetic variation"[All Fields] OR ("Variations"[All Fields] AND "Genetic"[All Fields]) OR "variations genetic"[All Fields]) OR "variations genetic"[Title/Abstract] OR ("genetic variation"[MeSH Terms] OR ("Genetic"[All Fields] AND "Variation"[All Fields]) OR "genetic variation"[All Fields] OR ("Variation"[All Fields] AND "Genetic"[All Fields]) OR "variation genetic"[All Fields]) OR "variation genetic"[Title/Abstract] OR ("genetic variation"[MeSH Terms] OR ("Genetic"[All Fields] AND "Variation"[All Fields]) OR "genetic variation"[All Fields] OR ("Diversity"[All Fields] AND "Genetic"[All Fields]) OR "diversity genetic"[All Fields]) OR "diversity genetic"[Title/Abstract] OR ("genetic variation"[MeSH Terms] OR ("Genetic"[All Fields] AND "Variation"[All Fields]) OR "genetic variation"[All Fields] OR ("Diversities"[All Fields] AND "Genetic"[All Fields]) OR "diversities genetic"[All Fields]) OR "diversities genetic"[Title/Abstract] OR ("genetic variation"[MeSH Terms] OR ("Genetic"[All Fields] AND "Variation"[All Fields]) OR "genetic variation"[All Fields] OR ("Genetic"[All Fields] AND "Diversities"[All Fields]) OR "genetic diversities"[All Fields]) OR "genetic diversities"[Title/Abstract] OR ("genetic variation"[MeSH Terms] OR ("Genetic"[All Fields] AND "Variation"[All Fields]) OR "genetic variation"[All Fields] OR ("Genetic"[All Fields] AND "Diversity"[All Fields]) OR "genetic diversity"[All Fields]) OR "genetic diversity"[Title/Abstract]) AND ("mutate"[All Fields] OR "mutated"[All Fields] OR "mutates"[All Fields] OR "mutating"[All Fields] OR "mutation"[MeSH Terms] OR "mutation"[All Fields] OR "Mutations"[All Fields] OR "mutation s"[All Fields] OR "mutational"[All Fields] OR "mutator"[All Fields] OR "mutators"[All Fields] OR "Mutations"[Title/Abstract]) AND ("polymorphism, genetic"[MeSH Terms] OR ("Polymorphism"[All Fields] AND "Genetic"[All Fields]) OR "genetic polymorphism"[All Fields] OR ("Polymorphisms"[All Fields] AND "Genetic"[All Fields]) OR "polymorphisms

	genetic"[All Fields] OR "polymorphisms genetic"[Title/Abstract] OR ("polymorphism, genetic"[MeSH Terms] OR ("Polymorphism"[All Fields] AND "Genetic"[All Fields]) OR "genetic polymorphism"[All Fields] OR ("Genetic"[All Fields] AND "Polymorphism"[All Fields])) OR "genetic polymorphism"[Title/Abstract] OR ("polymorphism, genetic"[MeSH Terms] OR ("Polymorphism"[All Fields] AND "Genetic"[All Fields]) OR "genetic polymorphism"[All Fields] OR ("Genetic"[All Fields] AND "Polymorphisms"[All Fields]) OR "genetic polymorphisms"[All Fields]) OR "genetic polymorphisms"[Title/Abstract] OR ("polymorphism, genetic"[MeSH Terms] OR ("Polymorphism"[All Fields] AND "Genetic"[All Fields]) OR "genetic polymorphism"[All Fields] OR ("Gene"[All Fields] AND "Polymorphism"[All Fields]) OR "gene polymorphism"[All Fields]) OR "gene polymorphism"[Title/Abstract] OR ("polymorphism, genetic"[MeSH Terms] OR ("Polymorphism"[All Fields] AND "Genetic"[All Fields]) OR "genetic polymorphism"[All Fields] OR ("Gene"[All Fields] AND "Polymorphisms"[All Fields]) OR "gene polymorphisms"[All Fields]) OR "gene polymorphisms"[Title/Abstract] OR ("polymorphism, genetic"[MeSH Terms] OR ("Polymorphism"[All Fields] AND "Genetic"[All Fields]) OR "genetic polymorphism"[All Fields] OR ("Polymorphism"[All Fields] AND "Gene"[All Fields]) OR "polymorphism gene"[All Fields]) OR "polymorphism gene"[Title/Abstract] OR ("polymorphism, genetic"[MeSH Terms] OR ("Polymorphism"[All Fields] AND "Genetic"[All Fields]) OR "genetic polymorphism"[All Fields] OR ("Polymorphisms"[All Fields] AND "Gene"[All Fields]) OR "polymorphisms gene"[All Fields]) OR "polymorphisms gene"[Title/Abstract] OR (("polymorphic"[All Fields] OR "polymorphics"[All Fields] OR "polymorphism s"[All Fields] OR "polymorphism, genetic"[MeSH Terms] OR ("Polymorphism"[All Fields] AND "Genetic"[All Fields]) OR "genetic polymorphism"[All Fields] OR "Polymorphism"[All Fields] OR "Polymorphisms"[All Fields]) AND ("genetic therapy"[MeSH Terms] OR ("Genetic"[All Fields] AND "therapy"[All Fields]) OR "genetic therapy"[All Fields] OR "Genetic"[All Fields] OR "genetical"[All Fields] OR "genetically"[All Fields] OR "Genetics"[MeSH Subheading] OR "Genetics"[All Fields] OR "Genetics"[MeSH Terms])) OR (("polymorphic"[All Fields] OR "polymorphics"[All Fields] OR "polymorphism s"[All Fields] OR "polymorphism, genetic"[MeSH Terms] OR ("Polymorphism"[All Fields] AND "Genetic"[All Fields]) OR "genetic polymorphism"[All Fields] OR "Polymorphism"[All Fields] OR "Polymorphisms"[All Fields]) AND "Genetics"[Title/Abstract]) OR (("polymorphic"[All Fields] OR "polymorphics"[All Fields] OR "polymorphism s"[All Fields] OR "polymorphism, genetic"[MeSH Terms] OR ("Polymorphism"[All Fields] AND "Genetic"[All Fields]) OR "genetic polymorphism"[All Fields] OR "Polymorphism"[All Fields] OR "Polymorphisms"[All

	Fields]) AND ("genetic therapy"[MeSH Terms] OR ("Genetic"[All Fields] AND "therapy"[All Fields]) OR "genetic therapy"[All Fields] OR "Genetic"[All Fields] OR "genetical"[All Fields] OR "genetically"[All Fields] OR "Genetics"[MeSH Subheading] OR "Genetics"[All Fields] OR "Genetics"[MeSH Terms])) OR (("polymorphic"[All Fields] OR "polymorphics"[All Fields] OR "polymorphism s"[All Fields] OR "polymorphism, genetic"[MeSH Terms] OR ("Polymorphism"[All Fields] AND "Genetic"[All Fields]) OR "genetic polymorphism"[All Fields] OR "Polymorphism"[All Fields] OR "Polymorphisms"[All Fields]) AND "Genetics"[Title/Abstract]))
Embase	('rheumatoid arthritis'/exp OR 'arthritis deformans' OR 'arthritis, rheumatoid' OR 'arthrosis deformans' OR 'beauvais disease' OR 'chronic articular rheumatism' OR 'chronic polyarthritis' OR 'chronic progressive poly arthritis' OR 'chronic progressive polyarthritis' OR 'chronic rheumatoid arthritis' OR 'disease, beauvais' OR 'infantile rheumatoid arthritis' OR 'inflammatory arthritis' OR 'polyarthritis, primary chronic' OR 'primary chronic polyarthritis' OR 'rheumarthritis' OR 'rheumatic arthritis' OR 'rheumatic polyarthritis' OR 'rheumatism, chronic articular' OR 'rheumatoid arthritis') AND ('microrna'/exp OR 'mirna' OR 'mirnas' OR 'micro rna' OR 'microrna' OR 'micrornas' OR 'genetic polymorphism'/exp OR 'genetic polymorphism' OR 'polymorphism (genetics)' OR 'polymorphism, genetic' OR 'gene mutation'/exp OR 'gene mutation' OR 'mutation, gene') AND ('das28'/exp OR '28 joint disease activity score' OR 'das28' OR 'disease activity score 28' OR 'disease activity score in 28 joints' OR 'simplified disease activity index'/exp OR 'sdai score' OR 'simple disease activity index' OR 'simplified disease activity index' OR 'health assessment questionnaire'/exp OR 'haq score' OR 'health assessment questionnaire' OR 'clinical disease activity index'/exp OR 'clinical disease activity index') AND ('case control study'/exp OR 'case control study' OR 'case-control studies' OR 'case-control study' OR 'control study, case' OR 'matched case control' OR 'matched case control studies' OR 'matched case control study' OR 'cohort analysis'/exp OR 'analysis, cohort' OR 'cohort analysis' OR 'cohort fertility' OR 'cohort life cycle' OR 'cohort studies' OR 'cohort study' OR 'fertility, cohort' OR 'phase 3 clinical trial'/exp OR 'clinical trial, phase 3' OR 'phase 3 clinical study' OR 'phase 3 clinical trial' OR 'phase 3 study' OR 'phase 3 trial' OR 'phase iii clinical study' OR 'phase iii clinical trial' OR 'phase iii study' OR 'phase iii trial' OR 'phase 4 clinical trial'/exp OR 'clinical trial, phase 4' OR 'phase 4 clinical study' OR 'phase 4 clinical trial' OR 'phase 4 study' OR 'phase 4 trial' OR 'phase iv clinical study' OR 'phase iv clinical trial' OR 'phase iv study' OR 'phase iv trial' OR 'clinical trial'/exp OR 'clinical drug trial' OR 'clinical trial' OR 'major clinical trial' OR 'trial, clinical')

Table S2. Excluded studies and their reason.

Study	Reason for exclusion
Michelle J Ormseth (1)	The association between miRNAs and treatment response is not evaluated.
J.C. De la Cruz-Castillejos (2)	Abstract with insufficient data
Fatima Heinicke *et al*. 2021 (3)	The study did not include MTX non-responder individuals.
Giovanni Pallio *et al*. 2020 (4)	It is a review. No miRNA is analysed. Authors only included gene polymorphisms (SNPs) related with response to biological therapy in RA patients.
Marwa M. Abdelaziz *et al*. 2021 (5)	The study does not analyse miRNA146a as a predictor of treatment response.
G. Kádár *et al*. 2016 (6)	EULAR 2016 abstract. The relation between miRNA146a levels and risk of relapse is studied.
M.-K. Lim *et al*. 2018 (7)	EULAR 2018 abstract. Authors analysed association between a panel of miRNA and RA activity.
E. Schordan *et al*. 2016 (8)	EULAR 2016 abstract. Authors communicated that 2 panels of miRNA are useful in discriminating responders from non-responders along RA patients. Abstract brings insufficient data and no full-article from this communication has been published.
B. Jekic *et al*. 2016 (9)	Authors analysed whether length polymorphism in the DHFR gene is associated with methotrexate (MTX) efficacy and toxicity in RA patients. None miRNA was analysed.
Chen *et al*. 2017 (10)	Evaluate response to TwHF and not to DMARDs.
Yan Liu et al. 2022 (11)	The study does not analyse miRNA as a predictor of treatment response.
Lopez-Pedrera C et al. 2020 (12)	Abstract and the data collected in an article already included in the review.
Ciesla M et al. 2020 (13)	ACR Abstract with insufficient data.

1. Ormseth MJ, Solus JF, Sheng Q, Ye F, Wu Q, Guo Y, et al. Development and validation of a microrna panel to differentiate between patients with rheumatoid arthritis, systemic lupus erythematosus, and control subjects. Arthritis Rheumatol. 2018;70:2070.

2. De La Cruz-Castillejos JC, Barbosa-Cobos RE, Becerril-Mendoza LT, Lugo-Zamudio GE, Ramírez-Bello J, Matias-Carmona M, et al. Evaluation of variants in miR-146a, miR-196a-2 and miR-499 and their association with susceptibility for rheumatoid arthritis and its extra-articular manifestations. Ann Rheum Dis. 2017;76:1133.

3. Heinicke F, Zhong X, Flåm ST, Breidenbach J, Leithaug M, Mæhlen MT, et al. MicroRNA Expression Differences in Blood-Derived CD19+ B Cells of Methotrexate Treated Rheumatoid Arthritis Patients. Front Immunol. 2021;12.

4. Pallio G, Mannino F, Irrera N, Eid AH, Squadrito F, Bitto A. Polymorphisms involved in response to biological agents used in rheumatoid arthritis. Biomolecules. 2020;10(9):1–11.

5. Abdelaziz MM, Gamal RM, Khalifa F, Mosad E, Sadek R, Abd El Razik DI, et al. MicroRNA146a gene polymorphism in patients with rheumatoid arthritis and the relevant value with disease activity and extra-articular manifestations. Egypt Rheumatol. 2022;44(1):97–101.

6. Kádár G, Czibula Á, Szalay B, Nagy K, Pusztai A, Balog A, et al. Predictors of disease course after the discontinuation of biologic therapy in rheumatoid arthritis patients with long-term remission. Ann Rheum Dis. 2016;75:1007.

7. Lim M-K, Song J, Kim SA, Yoo J. MicroRNA-1915-3p in serum exosome is associated with disease activity of rheumatoid arthritis in Korea. Ann Rheum Dis. 2018;77:266.

8. Schordan E, Bilger G, Coq M, Danilin S, Mehdi M, Schumacher M, et al. MiRNA profiling using HTG-EDGESEQ platform predicts response to anti-TNF alpha therapy in rheumatoid arthritis. Ann Rheum Dis. 2016;75:228.

9. Jekic B, Vejnovic D, Milic V, Maksimovic N, Damnjanovic T, Bunjevacki V, et al. Association of 63/91 length polymorphism in the DHFR gene major promoter with toxicity of methotrexate in patients with rheumatoid arthritis. Pharmacogenomics. 2016;17(15):1687–91.

10. Chen Z-Z, Zhang X-D, Chen Y, Wu Y-B. The role of circulating miR-146a in patients with rheumatoid arthritis treated by Tripterygium wilfordii Hook F. Med (United States) [Internet]. 2017;96(20). Available from: http://dx.doi.org/10.1097/MD.0000000000006775

11. Liu Y, Jeon S-M, Caterina MJ, Qu L. miR-544-3p mediates arthritis pain through regulation of FcγRI. Pain. 2021;

12. Lopez-Pedrera C, Luque-Tevar M, Pérez-Sánchez C, Font P, Patiño-Trives AM, De La Rosa IA, et al. Circulating Biomolecules as Potential Biomarkers of Early and Establishedresponse to TNFi Therapy in Rheumatoid Arthritis Patients. Arthritis Rheumatol. 2020;72(SUPPL 10):4015–7.

13. Ciesla M, Kolarz B, Dryglewska M, Majdan M. FCER1G gene methylation and mir-106/miR-17 as a new potential epigenetic markers in rheumatoid arthritis. Ann Rheum Dis. 2020;79(SUPPL 1):1347–8.

Table S3. Evidence table

Short Reference	Study characteristics	Cohort description	Intervention-Comparision	Results	Comments	Study quality (SING scale)
Peng Cheng, 2020	Design:<br>- 24-weeks prospective observational study.<br><br>Objectives:<br>- To study changes in circulating miR-125a and miR-125b after IFX treatment, and their predictive	Number of participants / groups:<br>- 96 healthy controls and 96 active RA patients with clinical history of at least single/multiple csDMARDs treatment.	Experimental group treatment:<br>- In serum: detect the presence of miR-125a and miR-125b, and measure their expression by RT-qPCR.	Magnitude of effect (+ confidence intervals / *p*-value):<br>- Patients vs healthy donors: both miR-125a and miR-125b expression were elevated in RA patients compared to healthy donors (p<0.001).	- Final multivariant analysis concluded that miR-125b could predict a better clinical response to IFX in RA patients but not miR-125a.	2++

	potential of clinic response in RA patients.<br><br>Study date:<br>- January 2016 to December 2018.	- 18.8% of the patients had presented IR to bDMARDs other than anti-TNF.<br><br>- Glucocorticoids: patients underwent glucocorticoid therapy within one month were excluded.	- Clinical evaluation: Clinical response was evaluated according to EULAR criteria after 4, 12 and 24 weeks.<br><br>- Patients were classified as responders or non-responders according to EULAR criteria.	- Association of miR-125a and miR-125b with RA inflammation: basal miR-125a levels positively correlated with CPR (p<0.001, r=0.395) while miR-125b levels correlated with number of painful joints (p=0.004, r=0.292), swollen joints (p=0.009, r=0.266), ESR (p=0.001, r=0.333), CRP (p<0.001, r=0.515), and DAS28-VSG (p<0.001, r=0.414) in RA patients.<br><br>- Treatment response: after 24 weeks of IFX treatment 69 patients (71.9%) responded to treatment while 27 patients (28.1%) did not.<br>- miRNA expression present a decreasing kinetic: Relative expression of miR-125a at W0 1.978 (1.304-3.419), W4 1.669 (1.006-3.013), W12 1.316 (0.776-2.431) and W24 1.189 (0.636-2.275), p<0.001. Relative expression of miR-125b at W0 2.420 (1.578-3.695), W4 1.937 (1.095-2.603), W12 1.612 (0.798-2.338) and W24 1.417 (0.676-2.220), p<0.001	- Associated treatment to anti-TNF: MTX (50 patients, 52.1%), and LEF (46 patients, 47.9%).

				- Relative expressions were higher in responders than in non-responders both for miR-125a (*p*=0.009) and miR-125b (*p*=0.002).		
Bart V. J. Cuppen, 2016	Design:<br>- 12-months prospective observational study.<br><br>Objectives:<br>- To investigate miRNAs that predicts the response to anti-TNF in RA patients, and to replicate results obtained in two previous studies.<br><br>Study date:<br>- Discovery phase: June 2009 to October 2012.<br><br>- Validation phase: October 2012 to June 2015.	Number of participants / groups:<br>2 cohorts.<br>-Discovery cohort: 80 patients were selected from BiOCURA study. The cohort is composed by patients with the most extreme clinical response to ADA and ETN. According to EULAR criteria, after 12 months they were defined as responders (n=40, 20 ADA and 20 ETN) or non-responders (n=40, 20 ADA and 20 ETN).<br>-Validation cohort: The best responders (n=20, 10 ADA and 10 ETN) and the worst non-responders (n=20, 10 ADA and 10 ETN) were selected.<br>- 72,5% of patients were DMARDs-naïve, 27,5% had IR to at least one bDMARDs,	Experimental group treatment:<br>- A panel composed of 758 miRNAs was analysed in serum and then validated by RT-qPCR.<br><br>- Clinical validation: according to EULAR criteria patients were defined as responders and non-responders after 12 months of treatment. Furthermore, patients with early treatment interruption were defined as non-responders.	Magnitude of effect (+ confidence intervals / p-value):<br>- Discovery cohort (n=80): 4 miRNAs were differentially expressed between responders and non-responders. Expression of both miR-99a and miR-143 was associated with ADA responders, while expression of miR-23a and miR-197 were associated with ETN responders.<br><br>- Validation cohort (n=40): None of the miRNAs could predict the response to anti-TNF treatment (*p*>0.05).	None	2++

		- Glucocorticoids: 30% treated with glucocorticoids. ADA treatment group (Responders: 13%, Non-responders: 50%, *p*=0.01). ETN treatment group (Responders: 20%, Non-responders: 33%, *p*=0.25)				
Maria Luque-Tévar, 2021.	Design:<br>- Prospective, multicentre, longitudinal study.<br><br>Objectives:<br>- To develop algorithms that predict the response to anti-TNF treatment in RA patients.<br><br>Study date:<br>- Not specified.	Number of participants / groups:<br>- A total of 104 RA patients from 2 independent cohorts. All patients had an IR ≥2 DMARDs. All patients were naïve to TNFi treatment.<br><br>- Glucocorticoids: 92% of patients with corticosteroids.<br><br>- 29 healthy donors.<br><br>- Serum samples collected at 0 and 6 months of treatment.<br><br>- Patients' inclusion period lasted 48 months (4 years).	Experimental group treatment:<br>- Responders were defined according to EULAR criteria.<br><br>- Treatment guideline in both cohorts:<br>(1) Infliximab (3 mg/kg/day at baseline and weeks 2 and 6; thereafter every 8 weeks).<br>(2) Etanercept (50mg/week).<br>(3) Adalimumab (40mg every 2 weeks).<br>(4) Golimumab (50mg every month).<br>(5) Certolizumab (400mg at baseline and weeks 2 and 4, thereafter 200mg every 2 weeks).<br><br>- After 3 and 6 months of	Magnitude of effect (+ confidence intervals / p-value):<br>- Before treatment:<br>(1) Altered levels of 223 miRNAs: 137 upregulated and 86 downregulated.<br>(2) 5 miRNAs related to RA pathology: miR-106a-5p, miR-143-5p, miR-148b-3p, miR-199a-5p and miR-346.<br>(3) Expression of these miRNAs is altered in RA patients compared to healthy donors ($p<0.05$): miR-160a-5p, miR-148b-3p and 199a-5p are downregulated; miR-143-5p and miR-346 are upregulated.<br><br>- At 3 months of treatment:<br>·Good response: 35%.<br>·Moderate response: 32%.<br>·No response: 33%.<br><br>- At 6 months of treatment:	- High levels of miR-106a-5p are included in the predictive model as indicator of good response to anti-TNF.	2++

			treatment it was evaluated: (1) SJC. (2) TJC. (3) DAS28. (4) CDAI. (5) SDAI. (6) HAQ.	·Good response: 49%. ·Moderate response: 20%. ·No response: 31%. ·In responders, the miRNAs levels are restored to levels found in healthy donors.<br><br>- Predictive model of response to treatment in discovery cohort: ·Among molecular parameters of good response, the logistic model identified the high levels of miR106a-5p. ·Molecular predictive model of response to treatment: AUC=0.807. ·Mixed predictive model (molecular and clinical variables) of response to treatment: AUC=0.909.<br><br>- Predictive model of response to treatment in validation cohort: ·Molecular model: AUC=0.750. ·Mixed model: AUC=0.831.		
SB Krintel, 2015.	Design: - Doubled-blind with placebo.<br><br>Objectives: - To identify miRNAs as biomarkers of	Number of participants / groups: - 180 DMARD-naïve and steroid-naïve patients with	Experimental group treatment: - Treatment: Adalimumab (40mg) or saline every 2 weeks.	Magnitude of effect (+ confidence intervals / p-value): - EULAR response: differences between treatment groups (120 most expressed	- After multivariable analysis: (a) High expression de miR-886.3p with low expression of	1+

	response to adalimumab.<br><br>Study date:<br>- Not specified.	early RA (OPERA).<br><br>- Glucocorticoids: peroral glucocorticoids were not allowed. Swollen joints were injected with triamcinolone.<br><br>- Definition of responders and non-responders after 12 months of treatment according to ACR/EULAR criteria.<br><br>- A panel of 377 miRNAs were analysed.	- Clinical evaluation: EULAR, DAS28<2.6 and Boolean (ACR/EULAR) after 12 months.<br><br>- There are no differences between treatment groups (ADA Vs saline).<br><br>- Saline group: 80/91 patients completed the study.<br><br>- ADA group: 81/89 patients completed the study.	normalization, *p*=0.008; quantile normalization, *p*=0.003; and rank normalization, *p*=0.011).<br><br>- DAS28 response: no differences (120 most expressed normalization, *p*=0.071; quantile normalization, *p*=0.372; and rank normalization, *p*=0.570).<br><br>- ACR/EULAR response: no differences (120 most expressed, *p*=0.083; quantile normalization, *p*=0.141; and rank normalization, *p*=0.035).<br><br>- miRNA expression profile associated with good EULAR response EULAR: Low expression of miR-22, miR-23a, miR-28.3p, miR-152, miR-185, miR-221, miR-886.3p; high expression of miR-590.5p and miR-660.<br><br>- Relation between miR-22 and EULAR response: low expression of miR-22 associated with good EULAR response in ADA group, not in saline group.	miR-22 have a 95% probability of good EULAR response.<br>(b) High expression of miR-886-3p with high expression of miR-22 have a 65% probability of good EULAR response.<br>(c) This association only in ADA group.<br><br>(b) Others identified miRNAs must be validated as indicators of response to inhibitors of TNF.

				- miRNAs associated with DAS28 response: miR-22, miR-23b y miR-758.<br><br>- miRNAs associated with ACR/EULAR response: miR-210, miR-215 y miR-146b-3p.		
Jacob Sode, 2018.	Design:<br>- Doubled-blind with placebo.<br><br>Objectives:<br>- To identify in plasma miRNAs predicting response to ADA or ADA/MTX.<br><br>Study date:<br>- Not specified.	Number of participants / groups:<br>- 180 DMARD-naïve and steroid-naïve patients with early RA (OPERA).<br><br>- Glucocorticoids: peroral glucocorticoids were not allowed. Swollen joints were injected with triamcinolone.<br><br>- ADA/MTX group: 89 patients.<br><br>- MTX group: 91 patients.<br><br>- A panel of 91 miRNAs was analysed.	Experimental group treatment:<br>- Treatment: 40mg ADA or placebo every week in combined with MTX.<br><br>- ADA/MTX Group: 1 patient changed to generic ADA, 1 changed to IFX, 2 changed to ETN, and 2 patients stopped the biological treatment.<br><br>- MTX Group: 2 patients stopped MTX/Placebo treatment and followed with DMARD, and 1 patient started ETN treatment.<br><br>- Treatment aim was a DAS28 score less than 3.2 without swollen joints.	Magnitude of effect (+ confidence intervals / p-value):<br>- No difference between treatment groups in their clinical and demographic characteristics.<br><br>- ACR/EULAR remission rate higher in ADA/MTX group than MTX group at 3 months (43% vs 25%, p=0.01) and 12 months (47% vs 31%, p=0.04).<br><br>- Prior to treatment:<br>(a) ADA/MTX group. Univariable analysis: 11 miRNAs present potential association with remission at 3 months, and 25 miRNAs with remission at 12 months. Multivariable analysis: no association with remission at 3 months, while miR-27a-3p is	- miR-27a-3p is a potential predictive biomarker for ADA/MTX treatment response in early RA patients.<br><br>- Regarding MTX treatment, patients presenting lower levels of miR-22-3p prior to treatment and a decrease in miR-22-3p levels at 3 months have a better response to MTX treatment.<br><br>- Plasma profiles could be useful predictive biomarkers.	1+

		associated with remission at 12 months (*q*=0.06).<br><br>(b) MTX group. Univariable analysis: 10 miRNAs associated with remission at both 3 and 12 months. Multivariable analysis: No association.<br><br>- After 3 months of treatment:<br>(a) None miRNA level at 3 months is associated with remission at 12 months in neither treatment group.<br><br>(b) Level changes of miR-27a-3p at 3 months compared to baseline associated with remission at 12 months: miR-27a-3p levels decrease in responders while they increase in non-responders (regression coefficient=–0.56, standard error=0.14, $p=2\times10^{-4}$, $q=0.02$).<br><br>(c) Improvement in DAS28 is associated with both increase in miR-16-5p, miR-51a, miR-92a-3p, miR-10b-5p and miR-29a-3p, and decrease in miR-22-3p and miR-145-5p ($q<0.1$).	

(d) Changes in miR-16-5p and miR-22-3p are associated with ΔCRP, ΔSJC, ΔTJC, and Δ[patient global VAS] (p<0,05).

(e) MTX group:
- High pre-treatment values of miR-16-5p are associated with decrease in DAS28. Moreover, increasing values of miR-16-5p at 3 months are associated with a decrease in DAS28 between 3 and 12 months.
- miR-22-3p presents a direct correlation with DAS28: low pre-treatment values of miR-22-3p are associated with decrease in DAS28 and decreasing values of miR-22-3p at 3 months are associated with a decrease in DAS28 between 3 and 12 months.

- Treatment response predictive model:
(a) ADA/MTX group:
- Model using miR-19b-3p for response at 3 months: AUC=0.63.
- Model using 10 miRNAs (miR-146b-5p, miR-19b-

				3p, miR-27a-3p, miR-16-5p, miR-423-5p, miR-27b-3p, miR-23a-3p, miR-06a-5p, miR-29b-3p, and miR-17-5p) for response at 12 months: AUC=0.82.<br><br>(b) MTX group:<br>- Model using 4 miRNAs (miR-10a-5p, miR-27b-3p, miR-03a-3p, and miR-26b-5p) for response at 3 months: AC=0.80.<br>- Model using miR-145-5p for response at 12 months: AUC=0.63.		
Ankita Singh, 2019.	Design:<br>- 4-months prospective observational study.<br><br>Objectives:<br>- To analyse whether baseline levels of miR-132-3p, miR-46a-5p and miR-155-5p can predict the response to MTX in RA patients.<br><br>Study date:<br>- Not specified.	Number of participants / groups:<br>- 94 RA patients.<br><br>- DMARD-naïve or had received MTX > 6 months back and had not taken any other DMARD.<br><br>- Glucocorticoids: no info.	Experimental group treatment:<br>- 10mg MTX weekly with 2.5mg increment every two weeks until (a) DAS28<2.6 or (b) 25mg/week.<br><br>- Responders (EULAR criteria): 73 RA patients.<br><br>- Non-responders (EULAR criteria): 21 RA patients.	Magnitude of effect (+ confidence intervals / p-value):<br>- Correlation between baseline levels:<br>(a) hsa-miR-132-3p and hsa-miR-46a-5p (*r*=0.50, *p*<0.05).<br>(b) hsa-miR-132-3p and hsa-miR-155-5p (*r*=0.48, *p*<0.05).<br>(c) hsa-miR-46a-5p and hsa-miR-155-5p (*r*=0.65, *p*<0.05).<br>(d) DAS28 and hsa-miR-132-3p (*r*=0.22, *p*=0.036).<br><br>- No correlation between:<br>(a) DAS28 and baseline levels of hsa-miR-46a-5p.<br>(b) DAS28 and baseline levels of hsa-miR-155-5p.<br>(c) RF, ESR, CRP, age, gender,	- Baseline levels of hsa-miR-132-3p, hsa-mªR-146a-5p and hsa-miR-155-5p are potential biomarkers of MTX response.<br><br>- RA clinical variables do not predict the response to MTX.	2++

disease duration and y baseline levels of miR-132-3p, miR-46a-5p and miR-155-5p.

- Comparison of baseline miRNAs level between responders and non-responders:
(a) hsamiR-132-3p (−8.03 (0.70), −7.47 (0.85); p<0.05).
(b) hsa-mªR-146a-5p (−5.11 (0.88), −4.62 (0.90); p<0.05).
(c) hsa-miR-155-5p (−7.59 (1.07), −7.00 (0.72); p=0.002).

- AUC, sensitivity and specificity of each miRNA:
(a) hsa-miR-132-3p (0.756, 95% CI 0.64–0.87, p<0.05); Sensitivity: 79.2%; Specificity: 61.9%.
(b) hsa-mªR-146a-5p (0.760, 95% CI 0.65–0.87, p<0.05); Sensitivity: 78.1%; Specificity: 61.9%.
(c) hsa-miR-155-5p (0.728, 95% CI 0.61–0.85, p=0.002); Sensitivity: 69.9%; Specificity: 71.4%.

- Increased miRNA expression after treatment (4 months):
(a) hsa-miR-132-3p; fold change: 1.56; p<0.05.
(b) hsa-miR-146a-5p; fold change: 1.31; p=0.001.

				(c) hsa-miR-155-5p; fold change: 1.61; *p*<0.05.<br><br>- Increased expression in responders and non-responders:<br>(a) hsa-miR-146-5p: only increased in responders (fold change: 1.39; *p*<0.001).<br>(b) hsa-miR-155-5p: increased in responders (fold change: 1.61; *p*<0.05).<br>(c) hsa-miR-132-3p: increased in both responders (fold change: 1.65; *p*<0.05) and non-responders (fold change: 1.37; *p*<0.05).		
Marzena Ciechomska, 2018.	Design:<br>- Prospective observational study.<br><br>Objectives:<br>- To analyse whether serum levels of miR-5196 are biomarkers of response to anti-TNFa treatment in RA and ankylosing spondylitis (AS) patients.<br><br>Study date:<br>- Not specified.	Number of participants / groups:<br>- 35 individuals:<br>(a) 10 RA patients.<br>(b) 13 AS patients.<br>(c) 12 healthy donors.<br><br>- Previous bDMARDs are not declared.<br><br>- Glucocorticoids: No info.	Experimental group treatment:<br>- RA group: Etanercept, % (n) 70% (n=7) Adalimumab, % (n) 30% (n=3) Methotrexate, % (dose of treated patients) 100% (10–25 mg)	Magnitude of effect (+ confidence intervals / p-value):<br>- Expression of miR-5196 is elevated in RA compared to healthy donors (fold change: 4.71; *p*=0.0001). After 6 months of treatment, miR-5196 levels decrease but are still higher (fold change: 2.18; *p*=0.012).<br><br>- All treated RA patients show a decrease in miR-5196 expression (fold change: 2.16; *p*=0.024), mean DAS28 (6.77 vs	- miR-5196 is a potential biomarker for anti-TNFa treatment response.<br><br>- Study limitation: small sample size.<br><br>- Secondary search.	2+

				4.48, $p<0.0001$), but not RCP (p=0,67).<br><br>- Changes in miR-5196 levels directly correlate with changes in DAS28 (p=0.039, r=0.67), while changes in RCP do not correlate with changes in DAS28 (p=0.38, r=–0.30).<br><br>- Treatment response prediction:<br>(a) miR-5196, AUC: 0.87, CI=0.63–1.11, Sensitivity: 83%, Specificity: 100%, p=0.055.<br>(b) RPC, AUC: 0.83, Sensitivity: 83%, Specificity: 75%, CI=0.56–1.10, p=0.088.		
Carmen Castro-Villegas, 2015.	Design:<br>- 6-months prospective observational study.<br><br>Objectives:<br>- To study whether serum levels of miRNA are indicators of treatment response in RA patients.<br><br>Study date:<br>- Not specified.	Number of participants / groups:<br>- 95 RA patients:<br>·Collected in 24 months.<br>·Inadequate response, at least, to 2 DMARDs.<br>·Glucocorticoids: 62% of patients.<br>·Naïve to anti-TNFa treatment.<br><br>- Discovery cohort: 10 RA patients (40% with glucocorticoids).<br><br>- Validation cohort: 85 Ra patients (64.7%	Experimental group treatment:<br>- 3 treatment groups:<br>· Infliximab (3 mg/kg/day intravenous infusion at times 0, 2 and 6 weeks, and every 8 weeks thereafter): 55 patients.<br>· Etarnecept (25mg twice a week): 25 patients.<br>· Adalimumab (40mg every week): 15 patients.	Magnitude of effect (+ confidence intervals / p-value):<br>- Mean DAS28 at baseline: 5.67 (5.29-6.11).<br><br>- After treatment:<br>· 85 patients (90%) defined as responders.<br>· Improvement in several indicators: TJC, SJC, SDAI, and HAQ ($p<0.0001$).<br><br>- miRNAs expression:<br>·Panel of 84 miRNAs: 75 miRNAS upregulated and 9	- miR-23 and miR-223 may serve both as predictor and biomarker of response to anti-TNFα/DMARDs combination therapy.<br><br>- Secondary search.	2++

		with glucocorticoids).	- Time-points: baseline and after 6 months of treatment.<br><br>- Definition of responders and non-responders according to EULAR criteria (DAS28).	downregulated after treatment.<br><br>·hsa-miR-125b, hsa-miR-126-3p, hsa-miR146a-5p, hsa-miR-16-5p, hsa-miR-23-3p, and hsa-miR-223-3p significantly upregulated after treatment in responder individuals ($p<0.05$).<br><br>·Non-responder individuals show no significant differences in any miRNA.<br><br>·Changes in miRNA related to changes in DAS28: hsa-miR-146a-5p ($p=0.001$, $r=0.400$); hsa-miR-223-3p ($p=0.026$, $r=0.292$); and hsa-miR-16-5p ($p=0.013$, $r=0.310$).<br><br>·Changes in miRNA related to changes in CRP: hsa-miR-126-3p ($p=0.004$, $r=0.349$); and hsa-miR-23-3p ($p=0.003$, $r=0.360$).<br><br>·Changes in miRNA related to ESR: hsa-miR-146a-5p ($p=0.008$, $r=0.312$).<br><br>·Baseline miRNA levels as predictors of treatment response:<br>(a) hsa-miR-23-3p: AUC=0.77,	

				Specificity=86.4%, Sensitivity=62.5%. (b) hsa-miR-223-3p: AUC=0.73, Specificity=90.2%, Sensitivity=57.1%. (c) Combined: AUC=0.76, Specificity=91.5%, Sensitivity=62.5%.<br><br>· Variation of miRNAs levels as predictor of treatment response: (a) hsa-miR-23-3p: AUC=0.77, Specificity=77.6%, Sensitivity=62.5%. (b) hsa-miR-223-3p: AUC=0.86, Specificity=84.3%, Sensitivity=57.1%. (c) Combined: AUC=0.88, Specificity=84.7%, Sensitivity=62.5%.		
Duroux-Richard, 2014	Design:<br>- Prospective observational study.<br><br>Objectives:<br>- To evaluate whether miR-125b is a biomarker of RTX treatment response in RA patients.<br><br>Study date:<br>- Not specified.	Number of participants / groups:<br>- Healthy donor: 13 individuals.<br><br>- Patients: 48 RA patients with IR to at least 2 DMARDs (MTX and anti-TNF included) and DAS28≥4.5.<br><br>- Glucocorticoids: For one month or more before the start, every patient was given oral corticosteroids and did not receive any intra-	Experimental group treatment:<br>- RTX, 1000mg at days 0 and 15.<br><br>- Treatment duration: 3 months.<br><br>- Responders: 16 patients. Non-responders: 16 patients.	Magnitude of effect (+ confidence intervals / p-value):<br><u>- Blood samples:</u> Expression of miR-125b elevated in RA patients compared to healthy donors (*p*=0.026). 75% of patients presents elevated miR-125b.<br><u>- Serum samples:</u><br>·Expression of miR-125b higher in responders than in non-responders (0.36±0.26 vs 0.19±0.12, *p*=0.002).<br>·Elevated expression of miR-125b is associated	- miR-125b as a possible useful marker for predicting RTX treatment success.<br><br>- AUC analysis is not carried out.<br><br>- Small sample size.<br><br>- Patients with established RA.<br><br>- Secondary search.	2+

		articular steroid injections.<br><br>- Definition of responders and non-responders according to EULAR criteria.		with good response to RTX.		
Liu, Y 2019	Design:<br>- 24-weeks prospective observational study.<br><br>Objectives:<br>- To explore the correlation between miRNAs and response to anti-TNF treatment.<br><br>Study date:<br>- January 2015 to December 2017	Number of participants / groups:<br>- 92 RA patients treated with etanercept during 24 weeks.<br><br>- 19% had IR to bDMARDs<br><br>- Glucocorticoids: no info.<br><br>- 3 months prior to study no biologics treatment.	Experimental group treatment:<br>- PBMC: Total RNA isolation and RNA microarray<br><br>- Microarray results: 59 miRNAs are upregulated and 78 downregulated when compare responders and non-responders.<br><br>- Top 10 dysregulated miRNAs are validated by qPCR: miR-146a-5p upregulated and let-7a-5p downregulated.<br><br>- Clinical evaluation: DAS28 at week 0, 6, 12 and 24 according to EULAR criteria.<br><br>- Treatment response according to EULAR criteria: 60 responder patients.	Magnitude of effect (+ confidence intervals / p-value):<br>- Multivariant logistic analysis of predictive factors for treatment response:<br>· miR-146a-5p (*p*=0.011) y CRP (*p*=0.006) correlate with good clinical response to anti-TNF treatment.<br>· let-7a-5p (*p*=0.002) and "biologics history" (*p*=0.037) are predictive factors for worse clinical response to anti-TNF treatment.	None	2+

	32 non-responder patients.	
